# Dieting

**Published:** 1881-02

**Authors:** W. W. Hall


### DIETING.

BY DR. W. W. HALL.

OUT of what we eat every particle of blood is made; every mouthful of food taken makes that much more blood, and is used in no other way than to make blood. If, then, there is too much blood in the whole body, or in any part of it, causing disease or suffering, we begin at the very foundation, by refusing to make any more blood; by refusing to eat an atom; and as each grown person eats and drinks several pounds a day, it is readily seen that abstinence from food stops at once the supply of blood. This is the medical treatment which blind instinct prompts the brute creation to adopt. A sick animal will not eat, or, if at all, most sparingly; hence the little disease observed in the animal creation, dying, as it does, by violence or age.

If the supply of blood is stopped in a case of disease by abstinence from food, the whole amount in the body is necessarily diminished every second of a man's existence, by the ordinary laws of the system, because the glands of the body are in operation more or less actively every second of our being. Their office is to diminish the amount of blood passing through them, as above explained as to the liver. Among the glands are the kidneys; they pass off a pound or more every day; the liver another. The whole skin may be considered a gland, and it passes off from the body in twenty-four hours, in the shape of visible and invisible perspiration, another pound or two of matter; then there are other places of discharge, the ears run wax, the eyes run water, the nose runs quite an amount in twenty-four hours; hence, a man not eating or drinking anything for a whole day would necessarily weigh a pound or two or more less, all in the shape of fluids, and these fluids being abstracted from the blood, the volume of blood is thus diminished several pounds by natural operations, which we cannot prevent. Nature instinctively comes to our aid by actually helping us to a desirable result, by taking away our appetite, and, in some cases, making even the sight and smell of food nauseating. Thus is dieting made easy, nature paving the way to a cure. But man may aid nature in her attempts to cure; he can hasten the diminution of the quantity of blood in the system, as a means of relieving himself of disease, by MUSCULAR EXERCISE.

We have selected the two foregoing articles—almost at random—

from Dr. Hall's book, "HEALTH AT HOME," or "HALL'S FAMILY DOCTOR." We do not believe that any similar work was ever published that gave expression to important truths in language so forcible and so easily comprehended. In the 850 pages which the book contains are condensed all the essential facts relating to ordinary diseases, with the simplest and most approved plans of treatment. It is not to be wondered at that the book has had a larger sale among families than any similar work ever published in any country.

"I AM an independent voter, and I can't support you until I've seen your platform," she said as he finished proposing. A couple of hours later it dawned upon the young man's mind that she wanted to know the amount of his salary.

